# Developing co-funded multi-sectoral partnerships for chronic disease prevention: a qualitative inquiry into federal governmental public health staff experience

**DOI:** 10.1186/s12961-020-00609-6

**Published:** 2020-08-20

**Authors:** Lee M. Johnston, Laurie J. Goldsmith, Diane T. Finegood

**Affiliations:** 1 Faculty of Health Sciences, Simon Fraser University, 8888 University Drive, Burnaby, V5A 1S6 Canada; 2 GoldQual Consulting, Toronto, Canada; 3 Department of Biomedical Physiology & Kinesiology Fellow, Morris J. Wosk Centre for Dialogue, Simon Fraser University, Burnaby, Canada

**Keywords:** Chronic disease, Multi-sectoral partnerships, Tacit knowledge, Public health practice, Public Health Agency of Canada, Coding paradigm, Trust, Capacity

## Abstract

**Background:**

Multi-sectoral partnerships (MSPs) are frequently cited as a means by which governments can improve population health while leveraging the resources and expertise of the private and non-profit sectors. As part of their efforts in this area, the Public Health Agency of Canada (the Agency) introduced a novel funding programme requiring applicants to procure matched resources from private sources to support large-scale interventions for chronic disease prevention. The current literature on MSPs is limited in its applicability to this model of multi-sectoral engagement. The purpose of this study was to explore the experiences of Agency staff working with potential partners to develop programme applications, such that we might identify lessons from adopting this type of partnership approach.

**Methods:**

Semi-structured interviews were conducted with the 12 staff working in the MSP programme. Interviews were recorded, transcribed and analysed using thematic analysis. Preliminary themes were used to inform follow up focus-groups sessions. A second round of analysis was conducted guided by a coding paradigm focused on understanding process.

**Results:**

We identified “experiencing uncertainty” to be a central concept in participants’ accounts of the MSP process, related specifically to the MSP programme’s novel conditions, shifts that occurred in sectoral roles and demands for new capacities. In response, Agency staff employed strategies to clarify partner interests, build trust in inter-sectoral relationships, and support internal and partner capacity. Outcomes associated with this process include impacts on trust between the Agency and potential partners, a deeper understanding of other sectors, and programme adaptations and refinements to address challenges related to the programme model.

**Conclusions:**

The co-funding model employed by the Agency is a potentially popular one for government bodies wanting to leverage funding from private sector sources. Our study identifies the potential challenges that can occur under this model. Some challenges are related to addressing material conditions related to partner capacity, whereas other challenges speak to deeper and more difficult to address concerns regarding trust and alignment of motivations and interests between partners. Future research exploring the challenges associated with specific models of MSP engagement is necessary to inform approaches to addressing complex problems through collaborative efforts.

## Background

The complexity of chronic disease has prompted calls for an ‘all-of-society’ approach that engages partners from a variety of sectors to explore, develop and implement potential solutions [1, 2]. Multi-sectoral partnerships (MSPs) have been proposed as a key means by which governmental public health organisations can work with other sectors to develop coordinated and sustained action to improve population health while leveraging the resources, knowledge and expertise of the private and non-profit sectors [3–5]. A considerable body of literature evidences the range of ways in which MSPs can be structured to support varying levels of collaboration and resource exchange across sectors, from purely transactional to highly collaborative, co-productive efforts [6–9]. These divergent MSP designs also establish the context for how universal partnership concerns, such as trust and interest alignment, are experienced by those involved [5].

Much of the literature on the practice of multi-sectoral engagement for public health in North American settings relates to inter-organisational efforts operating outside of a grant-making context [10, 11]. In contrast, the Public Health Agency of Canada (hereafter referred to as the Agency) has developed and implemented the Multi-sectoral Partnerships to Promote Healthy Living and Chronic Disease programme [12] (hereafter referred to as the MSP programme), which situates multi-sectoral engagement within a grant-making approach. The Agency has reconceptualised its traditional, hands-off approach to funding non-profit organisations, shifting instead to a model that aims to leverage funding from the private sector in support of innovative and sustainable approaches to health promotion. Under the new model, the Agency has posted an open call for eligible organisations and individuals — broadly defined to include most sectors — to submit a Letter of Intent (LOI) outlining an intervention that addresses common risk factors for chronic disease, will demonstrate measurable results, and has the potential to be scaled up to other populations or settings within Canada. Additionally, Agency staff are invited to actively solicit applications, broker relationships between potential partners and offer assistance in developing interventions so that they meet the programme’s criteria. Successful LOI applicants are invited to proceed to a full proposal submission, at which point they must have met another criterion — the procurement of matched funding from the private sector or other non-taxpayer-funded sources. The design of the MSP programme has supported the development of a diverse range of activity, including comprehensive community-based chronic prevention programmes, national programmes aimed at improving physical literacy and reducing obesity among children and youth, and efforts to increase healthcare capacity. Past partners have also been diverse, having included private citizens, financial institutions, foundations, media corporations, community centres, universities, and for-profit businesses ranging from pharmaceutical corporations to sporting apparel companies. The total request for federal funding for each project must be between CAN$200,000 and CAN$5,000,000, with interventions lasting from 2 to 5 years — representing a significant increase in scale and complexity when compared to previously funded activities.

This study aims to understand the experiences of Agency staff working in the initial years of the MSP programme (approx. 2013–2016) such that we might theorise about the process of developing co-funded MSPs within a programme that retains elements of a grant-making approach. We expect that this study’s findings will prove informative for governmental organisations considering models that combine grant distribution with leveraged funding drawn from private sector partners — an approach that may prove increasingly popular under governmental fiscal constraints. We also seek to contribute to the evidence base of practitioner knowledge related to MSP brokering work, in this case conducted from a governmental perspective. We expect that our analysis will prove informative for organisations interested in the potential implications of adopting an approach similar to that taken by the MSP programme.

## Methods

### Study design

We employed qualitative description [13] to document the experiences of governmental staff as they navigated the proposal application phase of partnership development. We were interested in situating these experiences within their broader context – that of a newly launched programme mandated to innovate in chronic disease prevention while engaging private sector partners. We expected that these conditions might present unique experiences for Agency employees – for both those previously working in a hands-off grants administration role as well as those experienced in the collaborative (and well-documented) approaches generally employed by public health organisations.

### Data collection

All personnel engaged in brokering or developing partnerships within the MSP programme were invited to take part in semi-structured interviews about their role at the Agency and their experience in the MSP programme (approved by the Simon Fraser University Office of Research Ethics; # 2016 s0256). We specifically asked participants to arrive at interviews prepared to describe a positive and challenging experience about their work in developing partnerships. This approach was informed by Ambrosini and Bowman’s [14] approach to eliciting and documenting tacit knowledge through narratives, such that participants could communicate in a manner reflective of our natural tendency to frame our experience as stories that come to represent an organisation’s collective memory. Where possible, interviews were conducted in person at the Agency’s office to build rapport and situate participants in a familiar setting. Two interviews were conducted over the phone. Interviews were approximately 60 minutes long and oral consent to participate in this study was obtained from interviewees.

Follow-up focus group sessions were conducted to build on emerging themes drawn from the individual interviews. Participants were sent a brief guide prior to their focus group session with broad questions to prime their thinking. Focus groups were structured by organisational role to facilitate deep dives into issues related to key themes based on commonalities among work roles. Each focus group session was approximately 2 hours long and conducted at the Agency’s offices. Interview and focus groups were digitally recorded, transcribed verbatim, checked for accuracy and uploaded into NVivo qualitative data analysis software (QSR International Pty Ltd. Version 11, 2014).

### Data analysis

Data analysis occurred in two phases. Firstly, individual interviews were analysed in NVivo using thematic analysis [15, 16]. This process entailed familiarisation with the interviews as full texts, inductive open coding and subsequent higher-level coding to identify recurring themes in the data. This process was led by one analyst (LMJ) with a second analyst (LJG) partially coding the data set, such that they could discuss the codes and their interpretations in detail. A third analyst (DTF) acted as an external arbiter at key phases throughout. Preliminary themes were used to inform questions for the semi-structured focus group sessions, which also served as an opportunity for MSP staff to speak to the accuracy of our initial interpretations. Focus group session transcripts were then integrated into the data set and coded using the same initial process. The results of the overall analysis were presented to the study participants during a project update to again assess the internal validity of the study findings through a member check.

During the second phase of analysis, two analysts (LMJ, LJG) adapted Strauss and Corbin’s [17] coding paradigm in order to further our understanding of the Agency’s process of developing co-funded multi-sectoral partnerships as situated in the context of the MSP programme. We re-examined the results of our thematic analysis through an iterative process that shifted between closer and more abstract configurations of our codes in order to identify the central analytic core of study participants’ experiences. Further use of the coding paradigm allowed us to identify the strategies employed in response to this central set of experiences and the outcomes that emerged from this process of experience and response. We also identified programme- and partnership-level factors that informed the contextual conditions in which those processes took place. Developing our model enabled us to explore relationships between these data points and consider actual and potential feedback mechanisms at play.

### Researcher relationship to the MSP programme

In order to document the lessons gained from the MSP programme, the Agency has developed a Learning and Improvement Strategy to support research inquiry into several facets of its approach. Due to our interest in multi-sectoral partnerships, our research team was connected with the Agency by a third party to discuss a study of staff experiences working within the programme. While the research team was involved in dialogue with MSP team members, we designed and executed our qualitative descriptive study independently from the Agency’s Learning and Improvement Strategy.

### A note about language

Over the course of this paper, we refer to those individuals and organisations with whom the Agency engaged as ‘partners’, ‘potential partners’ and ‘applicants’, depending in part on which aspect of their work is under discussion. That these terms can be used interchangeably reflects the fluid and complex nature of the relationships under discussion, as will be described in more detail further on. We also occasionally employ the term ‘case file’ — language also used by Agency staff — to describe individual instances of partnership and proposal development.

## Results

All personnel invited to participate in this study elected to do so. Upon completing the individual interviews, it was determined that one participant’s work experience did not meet the study criteria; the interview was excluded from analysis and the individual was not invited to attend the focus group sessions. At the time of individual interviews, the remaining 12 participants held primary job titles of policy analyst, grants and contributions administrator, or manager. Ten interviewees participated in focus groups (management focus group, *n* = 3; grants and contributions focus group, *n* = 4; policy focus group, *n* = 3). The remaining two participants were unable to attend due to time conflicts. Interviewees had public health backgrounds in a variety of governmental settings and with varied years of experience, with several having also had previous experience working with or in non-profits. One interviewee also stated they had previous experience working in the private sector.

**Fig. 1:**
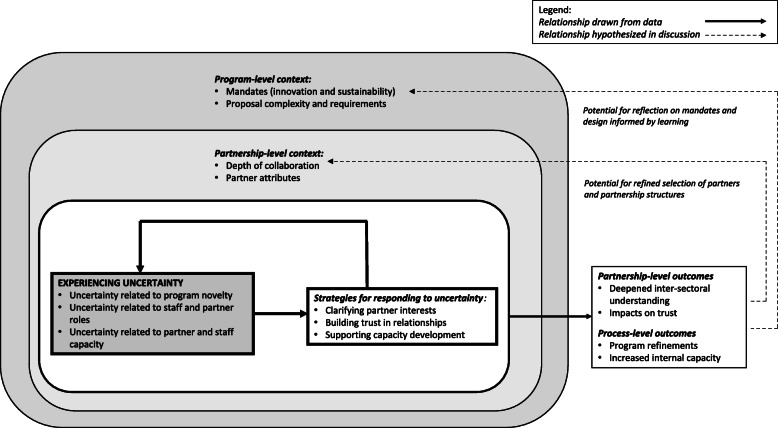
Experiencing uncertainty during the development of co-funded multi-sectoral partnerships

Figure 1 displays our model – an adaptation of the coding paradigm approach – and the categories expanded on in our results section, including our core category of experiencing uncertainty, the strategies employed in response to the experience of uncertainty, associated outcomes and relevant broader contextual factors.

### Experiencing uncertainty

Study participants’ accounts conveyed their experiences of uncertainty, or a lack of sureness, about various aspects of the MSP programme itself or interactions with partners within it. These experiences were informed by the broader, programme-level context established by the MSP programme’s complex design and mandates around fostering innovative practice and leveraging funds from private sources. They were also informed by the partnership-level contextual conditions unique to each case file, which represented various combinations of intervention type and complexity, organisational interests, stakeholder relationships, and depth of collaboration between the partners involved. Study participants identified high variation in the depth of their involvement in developing a proposal or intervention, with some arriving to the MSP programme ‘fully cooked’ and others needing significant input or resulting from collaborative, co-creative efforts. The uncertainty that emerged from these diverse conditions was related to three main areas, namely the ways in which the MSP programme presented a novel approach for federal governmental practice, the roles played by study participants and partners in the context of a co-funding partnership programme model, and concerns regarding capacity, both within the Agency and in applicant partners.

### Uncertainty related to programme novelty

The MSP programme’s mandate to address upstream determinants and shift away from a disease-specific prevention approach enabled study participants to reach out to stakeholders working in non-traditional intervention areas. While staff were excited about the opportunities afforded by the exploratory nature of this work, they also reflected on the overwhelming scope of their expanded mandates, as noted here:

*“Where I used to work, there was a cancer program, there was a diabetes program. So you were very, very focused in terms of subject matter. And now we are like, ‘healthy living’ is just – boom. It is all over the map.”*

Staff expressed uncertainty regarding their lack of content expertise in areas that fell outside of their previous work experience, such as the built environment, and noted the investment of time required to familiarise themselves with unfamiliar stakeholder landscapes. Staff also noted the risks or ‘potential minefields’ associated with interfering with those established stakeholder networks in which trust and mutual understanding had been established over time.

The MSP programme’s efforts to leverage non-governmental financial resources in the form of a matched funding requirement also marked a significant shift in approach for federal public health. Staff noted several instances in which applicants had difficulty procuring matched funding, resulting in frustrating situations where otherwise completed applications lay in an uncertain limbo until this requirement was met, as described here:

*“They don’t find the money. They don’t find matched funding. It comes down to as simple an issue as that, and that is always tough.”*

Study participants noted the dilemma the MSP programme helped to create by allowing applications to be developed without matched funding in place until its latter stages and reflected on how their deepening relationships with collaborative partners during the development process compounded tension. As one study participant noted:

*“You kind of develop that relationship as they are trying to build and find their private partner base, and it is really hard… It is almost like we have created this problem.”*

Working with private sector partners posed another source of uncertainty for Agency staff, the majority of whom were more familiar and comfortable with the non-profit sector and its mandates. Here, a study participant reflects on their experience with a novel sector:

“[The private sector partner] *said make sure you include a return on investment and analysis and that is when you know you are talking to a totally different sector.”*

Private partners varied in size and type with examples including individually owned and operated small business, pharmaceutical corporations, financial institutions, and food and beverage partners. While study participants identified tobacco and alcohol as being clearly off-limit private partners, the boundaries were less defined around engaging with other potentially controversial partners such as those in the food and beverage sector. Interviewees described learning about the private sector’s communication styles, motivations and trustworthiness as they came across inter-sectoral differences in approaches.

The MSP programme also established open timelines and a flexible work environment that introduced a type of procedural uncertainty that differed from traditional government ways of working. Staff noted the marked difference between this state of continuous decision-making and problem-solving without strict guidelines, often over lengthy periods of time, to the former traditional hands-off grants and contribution approach with clearer criteria for decision-making and fixed intake dates. Staff also identified the MSP programme’s ‘permissive’ culture and comfort with risk as being novel for a federal public health setting. Study participants employed the metaphor of ‘working in the greys’ to describe functioning in a continuous state of uncertainty, particularly in the MSP programme’s early days when guidelines and requirements were still in development. Study participants reflected on the exploratory and unique nature of the MSP programme, as in here:

*“I mean we are allowed to cold call people. You have to remember, where we came from* [in federal government]*, it was a fundamentally completely different environment.”*

*“We are covered in terms of the authority to do this work, but there is no bible that you can reach over the side of your desk and say, ‘okay, this is how you do that’.”*

Staff noted that, while this open and flexible approach enabled them to respond to and pursue emerging opportunities, it sometimes complicated relations with applicant organisations, as noted here:

*“Sometimes I find it a little hard, because it feels sometimes like rules change….I feel like that also poses some challenges for me because in working with partners, you need to be a bit clear about what the rules are.”*

Staff also noted that passing the LOI phase and entering into proposal development with Agency staff playing an active role in working with applicants implied a kind of tacit approval of the project — a potential source of problems given the uncertainty of the proposal’s eventual success. In this sense, the MSP programme’s timelines, while fostering staff’s ability to take advantage of emergent opportunities, could introduce the risk of increasing applicants’ expectations based on the Agency’s joint investment in a project’s success.

### Uncertainty related to staff and partner roles

Staff expressed some doubt about the nature of their role in the MSP programme, particularly between shifting between the partner and funder identities. For staff with a background in grants and contributions work, shifting from a hands-off administrative and oversight role to acting as a collaborative partner caused some discomfort, as noted by this study participant:

*“I like the idea of thinking of ourselves as partner brokers and helping building these partnerships, and I do believe in our model, but I still have a hard time wrapping my head around the idea of us being a partner at the table.”*

Other study participants struggled with assuming the role of partner in a context of a funding programme, as conveyed here:

*“There are power dynamics at play, so this is very different than for example, for me when I have been in a community, and I meet with sort of several organizations to say, ‘Let’s roll up our sleeves, and let’s think about how we can address this really challenging and complicated project together’.”*

*“For me it is funny because we always talk about each other being a project partner, and I still struggle with that. I still think that we hold the upper hand, because they are coming to us and asking us for funding, and we are saying yes or no…and we can still be fairly directive in that sense.”*

Tension in the meshing of a partnership and funding model also played out in instances where Agency staff were met with resistance from applicant organisations. A recurring dynamic was one in which applicants seemed to have difficulty ‘getting it’, in that they failed to respond to and incorporate staff suggestions on how to tailor their proposal to align with the programme’s mandates and requirements, as illustrated by these comments:

*“They came back to us three times. Either they couldn’t listen or didn’t hear us. Didn’t want to listen or didn’t hear us in terms of what we needed to ensure that there was a goodness of fit around our priorities, and the criteria for our funding program.”*

*“Maybe we don’t understand exactly what they are saying and that is kind of a communication side, but it is frustrating.…I think what we can see is the opportunity ahead and other people come to us sometimes with their really set ideas, and our job is to kind of help them shape it to fit our program but sometimes you just can’t.”*

Some staff associated applicant unwillingness to engage with Agency feedback with a broader resistance to, or failure to evolve with, the Agency’s efforts to shift traditional partner–funder dynamics and lessen dependence on government funding. Study participants also identified situations in which applicants’ receptivity to staff suggestions posed its own set of concerns:

*“The ones I really worry about are the small community groups who really need that money, and they will do anything to get our money, which means sometimes they promise things in their proposal that they can’t make, because they were just doing it to try and fit our model....They will bend over backwards.”*

Other situations occurred in which staff encouraged partner organisations with similar ideas to submit a joint application. Here, a study participant reflects on the downside of these unions:

*“We do have some instances where we kind of brokered a forced marriage in a way, because we wanted certain aspects to be included in our project. And those forced marriages haven’t necessarily gone that well, because there hasn’t been that kind of from the ground up development of collaboration and trust.”*

The depth of an applicant’s financial need and the funding–recipient dynamic were mediating factors in establishing trust and introduced uncertainties into collaborative engagement. Staff contrasted this experience to that of working with co-funder partners, some of whom provided more money to a partnership than did the Agency. These situations prompted their own shifts in government thinking about partnership work, as conveyed here:

*“When you are trying to bring the partner who doesn’t need the money, that is where the relationship feels incredibly horizontal if not inverse, because we are working harder to bring them in. We are trying to convince them to work with us and that is a different sort of relationship than what we have traditionally had with not-for-profits.”*

### Uncertainty related to partner and staff capacity

Insufficient capacity was identified as a source of uncertainty around organisations and individuals who struggled to convert a partnership or idea into an acceptable proposal. Study participants reflected on the implications that the MSP programme’s demands regarding evaluation planning, the development of indicators for measurable outcomes and pay-for-performance model had for partner organisations. As one staff member noted:

*“Our research proposal requirements are extensive and onerous, and in order to handle that, it actually really speaks to capacity. We don’t do this to test people’s capacity. Of course not. But it does.”*

Staff cited instances in which individuals, particularly those without strong organisational support, struggled to master the proposal requirements or to work within the extended timelines of the MSP programme, as noted here:

*“This person was just not emotionally capable of withstanding the sort of the roller coaster ride a bit, would get very, very frustrated with sort of the back and forth. Really couldn’t handle the back and forth.”*

*“It was clear to us that this person did not really understand* [the pay-for-performance] *concept, because of the questions we were getting back from them over and over again.*

*”*Staff noted that, while private partners brought their own unique capacities to partnerships and the interventions under design, their methods and focus for data collection and measurement did not necessarily align with the programme’s needs, as noted here:

*“When you are working with a non-traditional* [private] *partner, they have marketing metrics that I wouldn’t even begin to describe or understand. But what we need is data on reach, access and behavioural outcomes.”*

Staff also identified a lack of experience and knowledge in working with government as a capacity gap specific to private sector partners, as described here by a study participant:

*“Sometimes* [the private sector doesn’t] *necessarily have the same skills to deal with government relations in general….Most of my experience has been with* [non-profit organisations (NPOs)] *in the past and they usually have on staff a government relations person, who really understands the lay of the land, and how to navigate it and sort of different things that really need to be considered. My experience so far with the private sector is that they don’t always understand or fully understand all the implications of going in a certain direction.”*

For their part, Agency staff identified their own capacity needs as they navigated the novel conditions presented by the MSP programme. Investigating new intervention areas surfaced insufficient content expertise and the move into a more active partnership role was an exciting but uncertain transition for staff who lacked previous experience in partnerships. Additional needs around data collection and management, intervention design and communications emerged as the MSP programme became interested in co-developing potential projects that lay further outside of the Agency’s traditional comfort zone and broadened the scope of its portfolio in chronic disease prevention. Staff also noted their general unfamiliarity with the private sector, the relevance of which was dependent in part on the nature of the partnership structure and the private partner’s level of involvement. Interviewees worked to position themselves and the Agency’s mandates in relation to those of the private sector, as conveyed here:

*“The skills involved in working with the private sector are very different than working with an* [NPO whose] *whole reason of being is to help Canadians….Whereas if we are looking at the private sector, the whole reason for being is profit. Yes, they might have a corporate social responsibility angle and I do believe there are people that are good people and want to do good things, but it is different, and the skills in navigating that relationship, I am not sure there is a course for that.”*

### Strategies for responding to uncertainty

Agency staff developed an array of strategies aimed at addressing the uncertainties experienced in their programme work, as represented in the solid feedback arrows in Fig. 1. These strategies can be classified into three distinct but interdependent and reinforcing categories, namely clarifying partner interests, building trust in relationships, and supporting capacity development. Taken together, the strategies employed by Agency staff can be understood as efforts to foster alignment on issues both intangible and structural.

### Clarifying partner interests

Clarifying applicants’ and the Agency’s interests helped to ensure a common understanding as to what would be achieved through the intervention and how it would serve each organisation. Here, a study participant describes one such scenario of working with various partners to elicit their organisational needs and interests in regards to data outcomes on a particular intervention:

*“So then it was a matter of trying a few different approaches to surface the issue in all partner conversations to try and understand where the misunderstanding was….We have been asking, ‘What is the question, like what is the story that you want to tell? What is the question that we want to answer, so that you can tell the story that kind of meets your needs’. We are all going to tell different stories with this data, so it was trying to kind of surface it in a way that we could go, ‘okay we all understand’.”*

Taking time to surface partner needs and expectations helped Agency staff work toward a ‘goodness of fit’ between applicants and the MSP programme’s mandates and goals.

Surfacing and clarifying partner interests also served to address broader uncertainties regarding differences in inter-sectoral mandates. Agency staff described working with private sector partners to understand their motivations for investing in an intervention, without losing sight of the private sectors’ broader interests. One participant described this balancing act as such:

*“You can find the shared value in what we are doing together… but if there is a way to make their profit work to our advantage, then selling running shoes, selling hockey sticks, that is an okay thing.”*

While assessing and weighing the MSP programme’s fit with broader private sectoral interests was part of an ongoing and evolving conversation within the Agency, clarifying the interests and motivations represented on their specific case files helped staff to identify more immediate conflicts of interest.

### Building trust in relationships

Staff identified trust in inter-personal and inter-organisational relationships as being invaluable to their work in developing proposals and partnerships. As one interviewee framed it:

*“Wherever you are located, government or in a community based organization, it really is about the relationship, the relationships matter a lot….I found wherever I have worked if you know people, and you have the relationship, and there is the trust developed and you feel like it is genuine, then it really, really goes a long way.”*

Trust served as a conduit to managing conflict, addressing doubts about motivations and acquiring information necessary for accurately assessing partner capacity. Interviewees pointed to the different ways in which trust manifests itself in their work, noting, for example, that pre-established trust might inform their interactions with a potential partner, while the trust established between an applicant and upper management might not immediately establish itself in their own interactions with that potential partner. The ‘care and feeding’ of relationships was a cornerstone of staff practice, made manifest primarily through communication practices adapted to meet the needs of each individual case file and informed by the depth of collaboration required for each relationship within it. Staff also utilised a blend of individual and group communications, as noted by this study participant:

*“So we have partners — government partner and the private sector and the NGO. Sometimes it means a bilateral conversation, which then kind of influences how we are thinking, and then a trilateral call and then another bilateral call with this one. So it can be a series of conversations with not necessarily everybody together.”*

Communicating clearly, transparently and consistently with partners was deemed important to building trust and strengthening relationships. Doing so served to keep partners apprised of what was happening within the partnership where communication was not between all parties at the table, while also keeping partners up to date on their proposal’s status and any changes within the evolving MSP programme that might affect it. As one participant noted:

*“I have dealt with* [changes in the programme] *by just being clear with partners. You need to know that here at this point, this is how we work. That sometimes things change, and I will tell you what I know, and if there is sort of anything that is different, as we develop and then I will do that.”*

Lastly, Agency staff identified measures they had adopted in order to address potential barriers to trust in relationships, specifically around the imbalance of need between themselves and applicant organisations seeking funding. Included in this was clearly conveying the risks of participation in the MSP programme, as described by this study participant:

*“I usually am very up front with people when I will say, ‘This is what is required. It takes resources to write an LOI. And to develop a full proposal and look for matched funding partners, which are required at the time we submit your proposal for approval, is a commitment of time, and resources for which you won’t be compensated and for which there is not a guarantee at the end’. I am very candid about that.”*

In addition to communicating the risks associated with participating in the MSP programme, Agency staff also conveyed their appreciation of the value and assets that non-funding partners brought to partnerships. Some study participants framed this practice as a means of addressing potential power imbalances by acknowledging that, without implementing partners bringing their expertise and local connections to the MSP programme, it could not achieve its broader mandates.

### Supporting capacity development

Study participants embraced assisting applicants in accessing the skills, knowledge or expertise required to meet the MSP programme’s difficult requirements. This included educating applicants about unfamiliar concepts and increasing support in response to recurring challenges, as documented here:

*“I think one of the things that we have really changed in the way that we work is we provide a ton of support to the development of the intervention.”*

*“To ask someone to write a proposal, to find matched funding, to work in pay-for-performance—you can’t just insist that all these conditions have to be met when people haven’t worked in this way before. You* [have to be] *willing to provide them support, to explain what you mean, to have these very intrinsic conversations about what is a source of matched funding. What is a non-taxpayer funded source? How do you quantify in-kind?”*

Staff also connected applicants to academics or other specialists who could assist applicants in developing monitoring and evaluation plans, as described here:

*“What we need is data on reach and behavioural outcomes. So that is always a sort of extensive and extended discussion around capacity building, capacity understanding. So at that point I had suggested that we look to broker a relationship with a university based evaluator who would be able to support the type of data that we needed.”*

Another area in which applicants sometimes lacked experience was in navigating jurisdictional issues and policy landscapes, as in this example in which an interviewee describes helping an applicant struggling in unfamiliar territory:

*“We provided support. We were a sounding board. If they met some resistance, or you know there were some questions or sort of difficult situations, they would kind of come back to us, you know maybe, ‘What should we do next?’ So a bit of guidance and being there to help you and guide you, providing any knowledge that we have around the policy issue or the landscape.”*

Study participants also took measures to build capacity within the Agency, such as acquiring partnership brokering training and accessing resources within other governmental departments to support intervention development and design. While some gaps in capacity were addressed through formal measures, study participants emphasised the critical importance that experiential learning played in building their skills in partnership brokering and negotiating, working with new sectors and partners, and developing interventions that lay outside of the traditional scope of public health practice. An open flow of communication, a diversity of content expertise, and the combination of policy and grants and contributions experts on case files were all identified as factors enabling the exchange of knowledge and expertise within the Agency.

### Partnership- and process-level outcomes

Over the course of their accounts, study participants identified outcomes that emerged from their work in the MSP programme. These emerged in relation to two levels — the first being the partnerships themselves and relationships within them, and the second the broader level of the MSP programme.

### Partnership-level outcomes

Study participants identified a deeper understanding of the sectors with whom they engaged as a beneficial outcome from working closely and through complex interactions with programme applicants. Working collaboratively to understand each other’s interests, support each other’s capacity and invest time in the overall health of inter-personal and inter-organisational relationships all contributed to this phenomenon. In regards to the non-profit sector, this resulted in deepening Agency staff’s already high respect for the sector’s capacity and dedication to the public good, as noted here:

*“Now more so than ever, it amazes me what a non-profit can do with the resources that they are given, and how hard they work.”*

Deeper relationships with private sector partners resulted in a greater understanding of the sector’s motives and work styles and in appreciation for their marketing and data collection and management capacities. Given the lack of general experience that staff had had in working with private partners at the time interviews were conducted, this was an outcome still in development and largely informed by each interviewee’s experiences, as is reflected in this example:

*“We went to a sort of new level of mutual trust, and certainly with respect to this new private sector partner, I was blown away, and I felt, these guys really get it, like they completely understand this program. They are completely behind it.”*

Study participants also speculated that private sector partners’ understanding of government and its complexities had evolved, as noted here:

*“I find* [private partners] *are learning about how to work with government more, which I think it is good for them down the road. I think they are gaining some insight* [that we] *are not putting roadblocks just for the sake of roadblocks….I find they are learning and understanding about accountability that we have to the Canadian public.”*

This exchange of learning was further characterised by the high value that staff placed on the more deeply collaborative partnership arrangements that facilitated them. Here, staff reflect on the general value of active engagement:

*“We are certainly becoming more active partners than we were, certainly at the beginning of our process, and I think that we are starting to see just how valuable it is, and often for our partner organizations, they like it. They want us to be an active partner, because there is a reason they came to us, …not for just our money.”*

*“We don’t have just the relationship with the one organization that we are funding, but actually the private sector organization is very involved and we have a close relationship with the private sector organization….We are all one big team, and that is how we approached this, and to me that is a model of success.”*

Strong collaborative engagement presented a double-edged sword of risk and benefit. In addition to the deepened trust developed through inter-sectoral engagement, interviewees experienced that the negative outcomes of broken trust and risk to reputations and relations were acutely felt when breakdowns occurred. This was most present when the Agency ceased development on a project after a significant investment of resources by themselves and potential partners — a rare but critical incident for Agency staff in which their uncertainty about a proposal could not be addressed in spite of their efforts. Staff also identified the need to draw boundaries around their personal investment in relationships and the responsibility they felt toward applicants. As one study participant noted:

*“I care about the people and the projects, because I am a public health person by background. My heart is on my sleeve. I care about people and the work I do, but I still always have to keep this little guard up, right,* [in case] *things go sour, which they can.”*

### Process-level outcomes

Process-level outcomes were identified from staff’s reflection on the ways in which the MSP programme itself had evolved since its inception. Interviewees’ observations pointed to programme refinements in the form of more finely tuned processes as they pulled back from the more open and exploratory pursuit of potentially interesting ideas in favour of more selective decision-making about an intervention’s value to the programme. This shift was informed by factors such as incoming learnings from interventions already in the field as well as study participants adapting their practices in order to identify potential ‘deal-breakers’ earlier in the negotiation process, as suggested here:

*“The idea that indicators would drive my discussions, negotiations, collaborations, I wouldn’t have said that a few years ago; but really, it is a very clear way to see whether we can work together at all.”*

Study participants also spoke of the MSP programme becoming more selective about what partners might be best suited to the programme. In particular, staff differentiated between non-profit organisations who demonstrated a more favourable orientation towards the MSP programme’s mandates and goals and those non-profit organisations who seemed less likely to move away from their historical mandates and ways of operating, as reflected on by this interviewee:

*“Some* [not-for-profits] *are more ready and willing than others… Some take more time to come around to that and then there are a portion of them that haven’t been able to… You get a mix of all kinds from the* [NPO] *community, where some are willing to work, some are able to work, and then there are those who, there are those who can’t or won’t.”*

Finally, while study participants couldn’t speak to the longer-term effects of their capacity development efforts for partners, they did point to increased internal capacity and learning acquisition as outcomes of their early experiences in the MSP programme, as noted here:

*“I think in terms of my work, I feel like I have stretched myself a lot more. I feel like I know more. I can do more. I can handle more now.”*

*“I think as we learn more about these new types of projects, and these approaches* [to partnership]*, our internal capacity is growing, that we won’t need to rely on those intermediaries as much in the future.”*

## Discussion

Our study found uncertainty to be a central theme underlying federal governmental public health employees’ experiences as they developed multi-sectoral partnerships to support chronic disease interventions. The uncertainties we identified emerged in response to specific conditions established by the MSP programme, which in turn represented a shift from traditional governmental ways of working in terms of roles, inter-sectoral relationships, mandates, complexity and expectations. Our findings illustrate what the increasingly popular practice of multi-sectoral partnership working looks like specific to this programme and highlights potential uncertainties that implementers of similar approaches might expect to encounter.

One stream of our findings relates to procedural concerns emerging from the MSP’s proposal requirements, specifically regarding applicants’ capacity to develop interventions at the scale and technical specifications required under the MSP programme’s mandates. These findings reflect the proposition that individuals struggle in even modest levels of complexity, particularly when their capacity is incommensurate to the complexity of a task before them [18–20]. While they could be challenging and required an investment of time and resources, capacity gaps in regards to knowledge and technical expertise presented a relatively straightforward path for problem solving through information acquisition, knowledge exchange and the provision of structural support to struggling partners. The MSP programme’s conditions enabled this process, in that Agency staff were prepared and willing to play a supportive role for potential partners within open timelines that allowed this development to take place.

Our findings regarding the Agency’s interactions with the private sector echo common inter-sectoral uncertainties related to working with unfamiliar sectors, as Agency staff adapted to private sector cultural differences around performance measures, competencies, methods and pace of decision-making [5, 21]. The increased inter-sectoral understanding between government and the private sector noted in our findings also suggests that the MSP programme might be fostering the type of inter-cultural exchange that is frequently cited as a rationale for engagement with the private sector [4, 22]. Agency staff’s experience with the private sector was, however, very much in emergence given their relatively limited experience in comparison with the non-profit sector. The private sector partners described in interviewee accounts were also highly diverse in institutional scope, ranging from individually operated businesses to large multi-national corporations. This in turn presented conditions unique to each case file in regards to elements such as potential conflict of interest and power differentials. Our findings suggest the heightened value of experiential learning and the development of individual intuition and expertise in navigating interactions with new and diverse partnership pools, as the Agency continues to develop its approach to managing conflict of interest in this new arena.

When working with potential non-profit partners, Agency staff sometimes experienced tensions in identifying joint interests and finalising proposal details. Traditional thinking in inter-sectoral engagement posits that collaboration between government and the non-profit sector will be less complicated than with private partners — an assumption based on the long history of governmental and non-profit collaboration and their shared values regarding serving the public’s interests [23]. While this may be the case in other collaborative partnership settings, Agency staff’s experiences of difficult back and forth negotiation with non-profit partners lead them to question applicants’ motives. The dynamic identified between the Agency and some NPOs resembles cordial hypocrisy — a façade of congeniality that prevents honest communication and masks distrust and cynicism [24]. It may be reflective of a broader trend in which NPOs have conveyed their discomfort with funders’ push to partner despite mixed evidence as to its overall effectiveness [25–27]. Organisations’ natural tendency to view their own approach as being particularly needed — a trait that arguably makes achieving true shared responsibility in partnerships a relatively rare feat [28] — may also have contributed. Regardless of its source, our results suggest that the tensions experienced between the Agency and NPOs complicated two commonly accepted and related cornerstones of partnership work, namely the identification of aligned interests and the development of ‘genuine’ trust such as that cited by Agency staff as being present in their more successful relationships [5, 29, 30].

The challenges that emerged during these negotiations also speak to the implications of developing multi-sectoral partnerships for public health within a grant-making context. Theorists have identified multi-sectoral engagement as operating on a continuum ranging from one-way philanthropic funding dispersals to highly collaborative ventures in which organisations’ missions and activities begin to merge into collective action [26, 29]. The MSP programme’s design situates partnerships somewhere between these two ends of the spectrum, depending on the context of each case file. While purely transactional relationships may not require much inter-organisational dialogue to reach agreement, partnership studies suggest that, for more collaborative partnerships, the act of engaging in joint problem definition and establishing a course of action can be a key means for alignment interests and building trust. This process of developing a “*narrative coherence*” [25] about a problem and how to address it is also considered an important stabilising antecedent to successful collaboration [25, 26, 28]. The grant-making context, in which the Agency has final approval over funding allocation, requires projects to align with its organisational mandates and operates on a pay-for-performance structure, limits efforts to developing this narrative coherence in situations where organisations may be motivated to procure funding for their own activities. The uneven power differential that was also introduced by this context was acknowledged by Agency staff, in some instances challenging their identity as a collaborative public health practitioner. Their efforts to address these imbalances echo recommendations from the literature; namely, to provide resources to level the playing field, reassure partners as to the value of their role and engage in meaningful communication with partners at all phases of the process [25].

The grant-making context and its implications for multi-sectoral engagement also echo lessons found in the emerging literature on the context-specific dynamics of trust [10, 24, 31, 32]. Research suggests that, while pre-established trust can ease the partnership development process, changes in context such as those introduced by the MSP programme can exert a disproportionate effect on established relations [31]. Agency staff’s emphasis on nurturing and maintaining relationships is in keeping with the notion that trust is dynamic and receptive to external conditions and, as such, must be constantly cultivated in order to serve to reduce uncertainty, rather than become its source [24, 32]. It is also worth noting the important role that trust in inter-personal relationships played in Agency’s staff ability to navigate the challenges that emerged within the MSP development process. These relationships represent a form of affect-based trust — that is, trust emerging from an emotional bond that enables its participants to engage in leaps of faith above and beyond those supported by a more rational, cognition-based trust [31]. As such, it is a particularly valuable asset for the navigation of the complex inter-organisational relations that co-evolve with inter-personal relationships over time.

### Implications for practice

We propose the following takeaway messages for organisations interested in implementing a multi-sectoral partnership approach similar to that of the MSP programme. First, while the implementation of a more flexible approach situated within governmental broader mandates presented opportunities for Agency staff, it also introduced uncertainty for potential partners engaging with an evolving system. Clear and transparent communication, an adaptive approach to responding to emerging concerns and staff comfort with uncertainty were all necessary organisational characteristics for navigating multi-sectoral partnerships in these conditions. Second, organisations should anticipate the potential for resistance to government’s interest in being a more active partner, particularly from traditional funding recipients. Staff should be equipped to recognise and understand potential power dynamics as part of their partnership brokering skill set and be able to assess their implications for successful partnership development. Third, our findings suggest that organisations should consider playing a supportive, capacity-building role when changing their expectations of traditional partners, and be prepared to invest in internal capacity-building practices. Lastly, our findings also point to the value of investing time in building and maintaining relationships, given their potential to prevent other costly breakdowns during partner engagement. These takeaway messages also echo calls for repeat cycling with funding recipients as a means of maintaining and building upon the gains made through these aforementioned processes [27].

The outcomes identified in our findings suggest that Agency staff have reflected on the relative suitability of potential partners with the MSP programme and the value of engaging in higher forms of collaborative engagement. These findings suggest the potential for thinking strategically about partner and partnership structure selection (inner dotted feedback line, Fig. 1). The application of tools such as Austin and Seitanidi’s [33] collaborative value creation framework could contribute to a more nuanced and formalised identification of value potential in multi-sectoral engagement, above and beyond those associated with the interventions they support. This process could also further the evolution of broader programme mandates as they are reviewed in relation to the expected and actual outcomes emerging from multi-sectoral engagement.

### Implications for research

We echo the call of many researchers for a more robust research and learning agenda on the subject of multi-sectoral partnership development [5, 34, 35], specifically in relation to the value associated with a more developed typology of partnership engagement [4]. Developing this taxonomy could provide a critical unpacking of the many types of engagements that are frequently grouped under the term ‘partnership’, regardless of the level of interaction between players. Linking research that analyses the partnership development process in various programme contexts with the mid- and long-term outcomes of partnerships in practice would also contribute to a deeper understanding of partnership value in relation to the depth of collaborative engagement.

Our study also points to the value of conducting qualitative studies aimed at eliciting and documenting practitioner knowledge. We agree with the sentiment that complex problems and their policy responses require broader and more nuanced conceptualisations of evidence than is traditionally prioritised by government [36]. Given that tacit knowledge, or practice wisdom, can provide valuable information regarding the practical challenges and unintended consequences of policies and approaches in action, we see value in expanding and refining the application of qualitative methods for tacit knowledge capture.

### Limitations

This study is limited to presenting the perspectives of Agency staff only and does not represent the views of other participating partners.

## Conclusions

Multi-sectoral engagement with a co-funding component will likely become increasingly more popular with governmental agencies seeking innovative ways to address complex problems with limited resources. The MSP programme represents one of several potential approaches to engaging the governmental, not-for-profit and private sectors. This study documents some of the benefits and challenges associated with this approach as well as the strategies employed by Agency staff to address them. Our study points to the need for more context-specific studies of partnership development and the need to situate specific approaches to multi-sectoral engagement within the broad range of options available to governmental organisations.

## Data Availability

The data that support the findings of this study are not publicly available. Those interested in the data can contact the author for further information.

## Abbreviations

MSP: multi-sectoral partnership
LOI: letter of intent NPO non-profit organisation
